# Ascending aortic cannulation for Stanford type A acute aortic dissection

**DOI:** 10.1186/1749-8090-10-S1-A33

**Published:** 2015-12-16

**Authors:** Atsushi Iwakura, Yuki Hori, Satoshi Kono, Kazuo Yamanaka

**Affiliations:** 1 Wakayama Medical Center, Division of Cardiovascular Surgery, Wakayama, 6408558 Japan; 2 Tenri Hospital, Division of Cardiovascular Surgery, Nara, 6328552, Japan

## Background/Introduction

The real benefit of alternative cannulation technique for repair of acute type A aortic dissection remains controversial.

## Aims/Objectives

We evaluated our results of ascending aortic cannulation compared with femoral cannulation in acute type A aortic dissection.

## Method

From January 2000 to December 2014, 121 patients were operated on for acute type A aortic dissection. Cannulation was accomplished in 79 patients through the ascending aorta and 42 patients through the femoral artery.

## Results

Patient characteristics such as preoperative complications and shock status were almost similar between the groups. Although intraoperative parameters such as total cardiopulmonary bypass time, hypothermic circulatory arrest time, minimum bladder temperature and percentage of total arch replacement were also similar between the groups, ascending aortic cannulation significantly reduced arterial pressure on cardiopulmonary bypass circuit (156+39 vs 169+40 mmHg, p < 0.05), core-cooling time (33+10 vs 39+7 min., p < 0.01) and operation time (325+72 vs 357+96 min., p < 0.05). There were no significant differences in postoperative 30-day mortality and in-hospital mortality between the groups. However, incidence of temporary neurological dysfunction (4.0 vs 21.4 %, p < 0.05) and respiratory failure (4.0 vs 21.4 %, p < 0.05) as postoperative complications were significantly lower in the aortic cannulation group, although the incidence of stroke and renal failure did disclose any differences between the groups. Moreover, ascending aortic cannulation significantly reduced the hospital stay (26+10 vs 41+32 days, p < 0.01).

## Discussion/Conclusion

The ascending aortic cannulation for repair of acute type A aortic dissection can be advantageous to rapid cooling after the establishment of cardiopulmonary bypass. It might offer benefit on the incidence of postoperative complications and the length of hospitalization in patients with of acute type A aortic dissection.

